# Eavesdropping grey squirrels infer safety from bird chatter

**DOI:** 10.1371/journal.pone.0221279

**Published:** 2019-09-04

**Authors:** Marie V. Lilly, Emma C. Lucore, Keith A. Tarvin

**Affiliations:** Department of Biology, Oberlin College, Oberlin, Ohio, United States of America; Newcastle University, UNITED KINGDOM

## Abstract

When multiple species are vulnerable to a common set of predators, it is advantageous for individuals to recognize information about the environment provided by other species. Eastern gray squirrels (*Sciurus carolinensis)* and other small mammals have been shown to exploit heterospecific alarm calls as indicators of danger. However, many species–especially birds—emit non-alarm auditory cues such as contact calls when perceived predator threat is low, and such public information may serve as cues of safety to eavesdroppers. We tested the hypothesis that eavesdropping gray squirrels respond to “bird chatter” (contact calls emitted by multiple individuals when not under threat of predation) as a measure of safety. We compared vigilance behavior of free-ranging squirrels in the presence of playbacks of bird chatter vs non-masking ambient background noise lacking chatter after priming them with a playback recording of a red-tailed hawk (*Buteo jamaicensis*) call. Squirrels responded to the hawk call playbacks by significantly increasing the proportion of time they spent engaged in vigilance behaviors and the number of times they looked up during otherwise non-vigilance behaviors, indicating that they perceived elevated predation threat prior to the playbacks of chatter or ambient noise. Following the hawk playback, squirrels exposed to the chatter treatment engaged in significantly lower levels of vigilance behavior (i.e., standing, freezing, fleeing, looking up) and the decay in vigilance behaviors was more rapid than in squirrels exposed to the ambient noise treatment, suggesting squirrels use information contained in bird chatter as a cue of safety. These findings suggest that eastern gray squirrels eavesdrop on non-alarm auditory cues as indicators of safety and adjust their vigilance level in accordance with the vigilance level of other species that share the same predators.

## Introduction

Eavesdropping on public information to assess predation risk is common across a wide array of vertebrate taxa [1–4]. Exploitation of information about predator threat generated by other animals can benefit individuals by reducing the amount of time and energy they must allocate toward vigilance, while increasing the likelihood of avoiding predation. This in turn allows individuals to allocate more time and energy toward foraging and other tasks [1,4–8].

Public information about predation risk frequently takes the form of alarm signals [4], but non-alarm signals of safety also can be informative. For example, individuals of some group-living species act as lookouts and provide both alarm calls in the presence of danger and “sentinel” or “all-clear” calls in the absence of danger, such as when a predator leaves an area or following false alarms [7,9,10]. Like alarm calls, sentinel communications prompt adaptive changes in vigilance behavior from listeners [7,10–13]. Indeed, even the abrupt cessation of non-alarm vocalizations, as sometimes occurs in response to the sudden appearance of a predator, can elicit antipredator behaviors in eavesdroppers (e.g., [14]). Eavesdropping on each of these types of public information allows individuals to reduce their own costs of vigilance and in some cases they may rely on these environmental cues of predation risk [4,7,15].

Importantly, valuable public information may stem from heterospecific sources. For example, heterospecific alarm call recognition has been demonstrated in a wide array of species (e.g., [4]), and a variety of aquatic vertebrates and arthropods respond to chemical alarms generated by heterospecifics [16]. Though less frequently studied, a reduction of vigilance in response to heterospecific auditory signals or cues of safety has been experimentally demonstrated in at least 6 species (downy woodpeckers *Picoides pubescens* [5,9]; pied babblers *Turdoides bicolor* [17,18]; scimitarbills *Rhinopomastus cyanomelas* [19]; sociable weavers *Philetairus socius* [10]; dwarf mongooses *Helogale parvula* [20]; túngara frogs *Physalaemus pustulosus* [21]). Notably, in each of these studies, the responding species and the calling species exhibit tight ecological relationships–either moving together in foraging groups or occupying the same limited space during bouts of mate attraction–suggesting that the accessibility or reliability of public cues of safety may be dependent on frequent interactions between species. Moreover, in all but one of the studied cases (túngara frog), the calling species functions as an “information giver” or “community informant” [15,19] in that it is a particularly vigilant and vocal species within the foraging group, thereby providing copious accessible public information to eavesdroppers, as well as ample opportunity for eavesdroppers to evaluate the veracity of the information. As an example, Sullivan [9] found that downy woodpeckers responded to contact calls of nuclear species from the mixed-species flocks with which they frequently forage, but not to contact calls of sympatric species with which they rarely interact. However, we are unaware of any studies other than Sullivan [9] that have tested for eavesdropping on non-alarm cues of predation risk generated by heterospecifics that do not have a tight ecological relationship with the focal species.

If contact calls emitted by individuals that perceive themselves to be at low risk of predation is generally a reliable index of predation risk, then it seems reasonable to expect such information to be readily be exploited by heterospecifics that share a similar suite of predators, even if they do not have tight ecological relationships with the informant species. Given that possibility, here we test the hypothesis that gray squirrels (*Sciurus carolinensis*) exploit bird “chatter,” defined here as a collection of contact calls generated by multiple individuals, as a cue to predation risk because chatter is likely to be emitted by birds only when they perceive predator threat to be low [22]. We focus on chatter instead of contact calls emitted by a single individual because we assume that chatter represents more reliable information about predator threat: by definition, multiple individuals–each with their own eyes and ears–contribute to the din of chatter (e.g., [23]). Gray squirrels frequently occupy habitat which a variety of songbird species share or move through, but squirrels are not specifically attracted to, nor do they follow, bird flocks, and birds do not follow foraging squirrels. Thus, although the squirrels are sympatric with a variety of bird species, they do not have “tight” ecological relationships with them.

We primed foraging gray squirrels (*Sciurus carolinensis*) to an elevated risk of predation by presenting them with a playback recording of the call of a red-tailed hawk (*Buteo jamaicensis*), a predator of squirrels and one to which many songbirds respond with alarm calls [24,25]. We followed the hawk call with playbacks of either bird chatter or non-masking low amplitude ambient background noise lacking bird chatter and measured changes in squirrel vigilance over a 3-minute period. The hypothesis that chatter functions as a public cue of safety predicts that the heightened squirrel vigilance in response to the hawk call should be lower and decay more quickly in squirrels exposed to bird chatter than in those exposed just to ambient background noise.

## Materials and methods

### Study species and field sites

Gray squirrels forage in trees and on the ground in forest, forest edge, and parkland. When foraging and handling food, squirrels frequently pause and briefly scan their surroundings, presumably to survey for potential predators [26]. Although gray squirrels do not follow particular bird species while foraging, they share habitat with many songbird species (including mixed-species foraging flocks) in our study area. They also attend to alarm calls of American robins (*Turdus migratorius*; [27], black-capped chickadees (*Poecile atricapillus*) and tufted titmice (*Baeolophus bicolor*; Tarvin, unpublished data), and probably other species, and as well as to calls of blue jays (*Cyanocitta cristata*), a potential competitor that may kleptoparasitize their food caches [3]. We presented playback recordings to free-ranging gray squirrels throughout parks and residential areas in Oberlin, Ohio, USA. Squirrels in these areas were generally habituated to the presence of humans, allowing us to conduct playback trials at close distances without unduly disturbing them [27,28]. Nonetheless, we avoided sampling squirrels in parts of the study site that were heavily trafficked by humans to avoid disturbance during trials [28]. To avoid habituation of squirrels to the playbacks and resampling of individual squirrels, focal individuals were separated by at least 164 m (roughly, the home range of adult male eastern gray squirrels in woodland habitats [29]), and we did not conduct playback trials more than once at any site to avoid resampling the same squirrel [27].

### Playback recordings

We made stereo recordings of chatter from birds visiting a feeder in the absence of obvious predation threat approximately 24 km from the study site on the mornings of 19 and 25 January 2016 (two recordings, approximately 10 and 14 min each) and 09 Dec 2016 (one recording, approximately 23 min), using an Olympus LS-10 linear PCM recorder (Olympus Imaging America, Center Valley, PA USA). We did not detect predator calls or alarm calls on any of these recordings. In addition to contact calls, the chatter recordings included wing flutter noises, sounds of small birds hopping in dry leaves, distant low-amplitude traffic and river noise, and noises associated with an adjacent building. We made one exemplar cut containing at least three minutes of chatter noise without abrupt wind noise from each base recording, and thus used three unique exemplar recordings for the chatter treatment to reduce pseudoreplication [30]. Species present on the exemplar cuts are listed in Table 1. We made similar recordings of ambient background noise at the same feeder on the nights of 02 May, 18 Nov, and 21 Nov 2016. These “ambient noise” recordings therefore contained similar low amplitude building and distant traffic and river sounds from the same location, but no bird calls, wing flutter, or leaf noises. Again, we made one exemplar cut containing at least three minutes of ambient noise without abrupt wind noise from each base recording, and therefore used three unique exemplar recordings for the ambient noise treatment.

**Table 1:** Songbird species emitting contact calls and other sounds on the three chatter exemplar recordings used in this study. All vocalizations listed in the table are contact calls unless otherwise noted.

	Chatter_A (19 Jan 2016)	Chatter_B (25 Jan 2016)	Chatter_C (09 Dec 2016)^b^
**Songbird species present on recording**^a^	House finch Black-capped chickadee (distant song) Downy woodpecker Dark-eyed junco Northern cardinal	White-breasted nuthatch American goldfinch Dark-eyed junco White-throated sparrow Downy woodpecker American crow (distant)	White-throated sparrow House finch Tufted titmouse Dark-eyed junco
**Other noises**	Flutter noises Leaf noises	Flutter noises Leaf noises Tractor?	Flutter noises Leaf noises Distant traffic/river noise
**Additional comments**	Several individuals calling at once	Multiple individuals calling at once	Rarely more than one individual calling at a time

^a^ House finch *Haemorhous mexicanus*; Black-capped chickadee *Poecile atricapillus*; Downy woodpecker *Dryobates pubescens*; White-throated sparrow *Zonotrichia albicollis*; Dark-eyed junco *Junco hyemalis*; White-breasted nuthatch *Sitta carolinensis*; Northern cardinal *Cardinalis cardinalis*; American goldfinch *Spinus tristis*; American crow *Corvus brachyrhynchos*; Tufted titmouse *Baeolophus bicolor*

^b^ The base recording from 09 Dec 2016 contained calls of a blue jay (a potential kleptoparasite of squirrels [3]), but we took the exemplar cut from a section of this recording made several minutes before the jay began calling.

We obtained three exemplar recordings of red-tailed hawk calls from Xeno-Canto (Xeno-Canto catalog numbers: 34863, 321708, and 31160). Each hawk call exemplar was paired with both a chatter and an ambient noise exemplar to control for any abnormal information that might be encoded in the hawk call, making for 6 different playback exemplars in total [30,31]. Red-tailed hawk playbacks were presented at a sound pressure level of 75–80 decibels (measured 1 m from the speaker) as this falls within the natural sound level range of a hawk calling while hunting overhead [31,32]. Chatter playback was played at a sound pressure level of at least 40 decibels as terrestrial wildlife begin responding at this noise level [33]. The hawk call was played in mono from one speaker to mimic a natural call from a single hawk, while the chatter and ambient noise treatments were played in stereo to mimic natural bird chatter or ambient noise across a range of space. The order of playback exemplars was randomized before going out in the field.

### Experimental design

Upon encountering a squirrel that met the criteria for inclusion in the experiment (at least 164 m from the site of any previously conducted trial, no predators observed in the area), the observer (MVL in all trials) approached to within 15–30 m, set up speakers as quickly and inconspicuously as possible, and then moved at least 5 m away from the speakers so as to minimize association between her presence and the playback. Playback recordings were broadcast from an mp3 player (FecPecu Lossless Sound 8GB MP3 Player, IQQ/OEM, Guangdong, China) through two powered Fender Passport Mini speakers (Fender Musical Instrument Corporation, Corona, CA, USA) elevated 38 cm off the ground on inverted plastic buckets to reduce sound attenuation and spaced 4 m apart to achieve the stereo effect of the recordings. We waited at least 5 minutes after the cessation of any naturally occurring alarm calls before beginning a trial, though natural alarm calls only occurred prior to 2 trials. No natural alarm calls occurred during the treatment period. We followed this setup with a one-minute period of acclimation before beginning experimental trials.

Following the one-min acclimation period, trials comprised a “pre-hawk period” consisting of 30 seconds of silence (no playback), a 1–3 sec playback of a red-tailed hawk call, and a “post-hawk period” consisting of 30 seconds of silence. The post-hawk period was followed with a treatment period consisting of 3 min of either chatter or ambient noise playback. We measured squirrel vigilance during the pre- and post-hawk periods, and in three consecutive 60 sec segments during the treatment period.

We used focal animal sampling [34] to measure squirrel vigilance behavior. In our sampling design, squirrels could occupy any of six mutually exclusive behavioral states: foraging, preening, resting, standing, freezing, and fleeing. Foraging, preening, and resting constituted non-vigilant behavioral states, while standing, freezing, and fleeing constituted vigilant states. Foraging was designated when the squirrel was searching for food or eating, while preening was designated as scratching, licking fur, or other non-foraging maintenance behaviors. Resting was designated when the squirrel appeared relaxed and was not foraging or preening. Standing was designated when a squirrel reared up on its back legs, apparently to achieve an elevated visual perspective while scanning for predators [9,26]. Freezing was distinguished from resting when the squirrel abruptly tensed its body or flattened and ceased eating or preening, and fleeing was designated as abruptly ceasing its current behavior and running without pauses. Freezing and fleeing are common responses by tree squirrels to threat [26]. In addition to these six behavioral states, we recorded all instantaneous changes in position of the head (up, down, or to the side) that appeared to be momentary scans of the environment [9,26]. We treated these brief scans (hereafter, “lookups”) as vigilant behavioral events. This sampling protocol allowed us to generate an accurate representation of the proportion of time that a squirrel was engaged in vigilant behavioral states as well the frequency of vigilance events [34,35].

We recorded all behaviors using ClockWork, a webapp timer designed and customized specifically for the project [36]. The webapp included buttons with separate timers for each behavioral state and another button with which we recorded each lookup event with a timestamp, allowing us to record the number of seconds engaged in each behavioral state as well as the number and timing of lookups during each observation period. When the trial began, the button was pushed for the behavior type that the squirrel was engaged in, automatically creating a timestamp for the start of the trial. When the focal squirrel began engaging in a different behavior type, pushing the new behavior button automatically stopped timing the previous behavior, started timing the new behavior, and organized the behaviors by order in which they occurred.

Each trial focused on a unique squirrel, and each squirrel in the study received only one playback (either chatter or ambient noise). We presented trials to squirrels located in trees or on the ground, but noted the microhabitat in order to control for variation in vulnerability level of the focal squirrel and thus variation in the intensity of the vigilance response [27]. After each trial was completed, we measured or estimated to within 0.5 m the distance from the midpoint between the speakers to the squirrel [27,37].

All trials were conducted in the field by MVL to reduce inter-observer bias. MVL conducted multiple training trials prior to the onset of data collection to increase standardization of behavioral assessments.

### Data analysis

None of our dependent variables fit normal or Poisson distributions and the error distributions from generalized linear models were not normal; hence, we were unable to use generalized linear or mixed effect models to analyze the data. Therefore we used non-parametric tests (IBM SPSS Statistics, v 24) and permutation tests (program lmPerm in R [38,39]) for all remaining analyses. Permutation tests are much less sensitive to assumptions about underlying distributions than parametric tests, so are appropriate for our analyses [40].

We tested whether squirrel vigilance increased in response to the hawk call (but prior to treatment playback) by comparing levels of vigilance during the pre- and post-hawk periods. We used Wilcoxon’s matched-pairs signed-ranks test to assess the difference in the number of lookups between the pre- and post-hawk periods. Similarly, we used the same test to assess the difference in the number of seconds the squirrel was engaged in vigilant behavioral states (freezing, standing, fleeing) as opposed to non-vigilant states (foraging, preening, resting) during each time period.

To assess whether squirrels in the two treatments differed in their sensitivity to the hawk call, we tested whether the tendency to engage in vigilance differed between squirrels destined for the chatter treatment and those destined for the background treatment using permutation tests of a linear model with difference in number of lookups between pre- and post-hawk periods as the dependent variable and eventual treatment and habitat vulnerability as predictor variables. We coded habitat as ‘1’ for high vulnerability when the focal squirrel was in an open area such as on the ground or a fence, and ‘0’ for low vulnerability when the squirrel was in a tree. We similarly tested for a relationship between change in percent of time spent vigilant before and after presentation of the hawk call and eventual treatment while controlling for habitat vulnerability.

We tested for an effect of chatter on overall vigilance level during the 3 minutes following the post-hawk period using permutation tests of linear models. Our models tested whether treatment predicted vigilance level during the treatment period (i.e., number of lookups or time spent vigilant, respectively) while controlling for habitat vulnerability and vigilance level prior to the onset of the treatment (i.e., during the 30 sec post-hawk period). We also tested whether the decline in vigilance following the hawk call differed between treatments by predicting the change in vigilance during the treatment period while controlling for habitat vulnerability and vigilance level during the post-hawk period. We calculated change in number of lookups as the number of lookups per 30 seconds during the third minute of the treatment period minus the number of lookups during the 30-sec post-hawk period. We similarly calculated change in percent of time spent vigilant as the percent vigilance during the third minute of the treatment period minus the percent vigilance during the 30-second post-hawk period.

The use of animals in this study was approved by the Oberlin College Institutional Animal Care and Use Committee, IACUC protocol F15RBKT-1.

## Results

We conducted 30 trials with the chatter treatment, and 37 trials with the ambient noise treatment. Because squirrels sometimes disappeared from view before a trial was completed, we obtained 3 full minutes of data from the post-hawk period for 28 chatter trials and 26 ambient noise trials. Thus, the sample size for analysis of response to the hawk call was 30 chatter trials and 37 ambient noise trials, and the sample size for all other analyses was 28 chatter trials and 26 ambient noise trials.

### Response to hawk call

**Fig 1:**
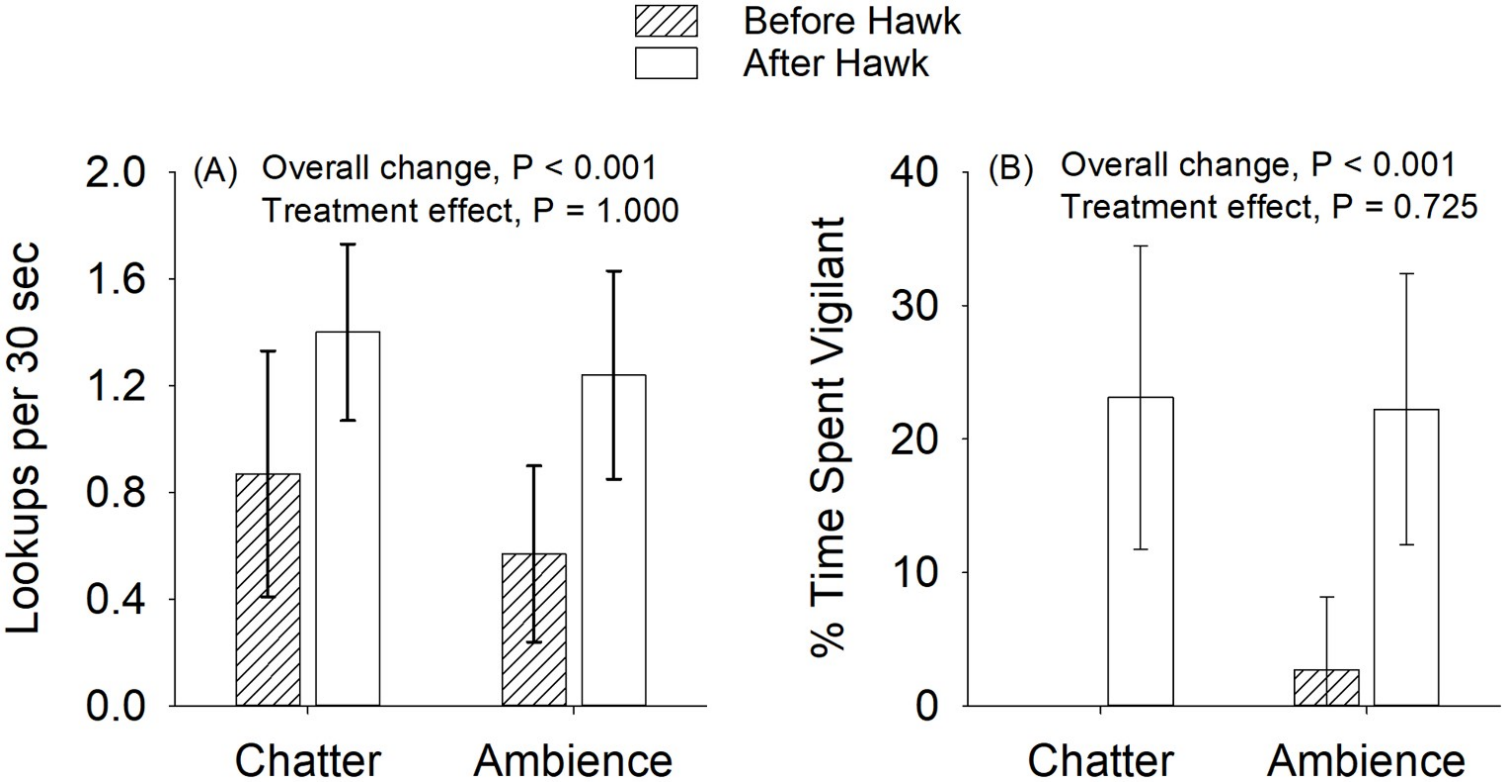
Change in vigilance behavior of squirrels in response to playback of a red-tailed hawk call. Change in vigilance behavior of squirrels in response to playback of a red-tailed hawk call, partitioned by the treatment to which squirrels were eventually exposed. Error bars represent 95% confidence intervals. Means are not adjusted for effects of habitat vulnerability. The mean value for % Time Spent Vigilant prior to the hawk call for squirrels eventually exposed to the chatter treatment was 0, with no variance.

Squirrels responded to the hawk call by significantly increasing number of lookups (Wilcoxon Signed Ranks Test, Z = -4.281, P < 0.001) and time spent vigilant (Wilcoxon Signed Ranks Test, Z = -4.783, P < 0.001) (Fig 1). We found no difference between treatment groups in the change in number of lookups or time spent vigilant in response to the hawk call prior to the onset of the treatment playback when controlling for habitat vulnerability (change in lookups: treatment estimated slope = -0.04, iterations = 51, P = 1.000, vulnerability slope = -0.75, iterations = 575, P = 0.15; time spent vigilant: treatment slope = 1.90, iterations = 51, P = 0.725, vulnerability slope = -3.15, iterations = 63, P = 0.619; Fig 1). Thus, squirrels in the chatter and ambient noise treatment groups did not differ in their tendency to engage in vigilance behaviors in response to the hawk call.

### Difference between chatter and ambient noise treatments

**Fig 2:**
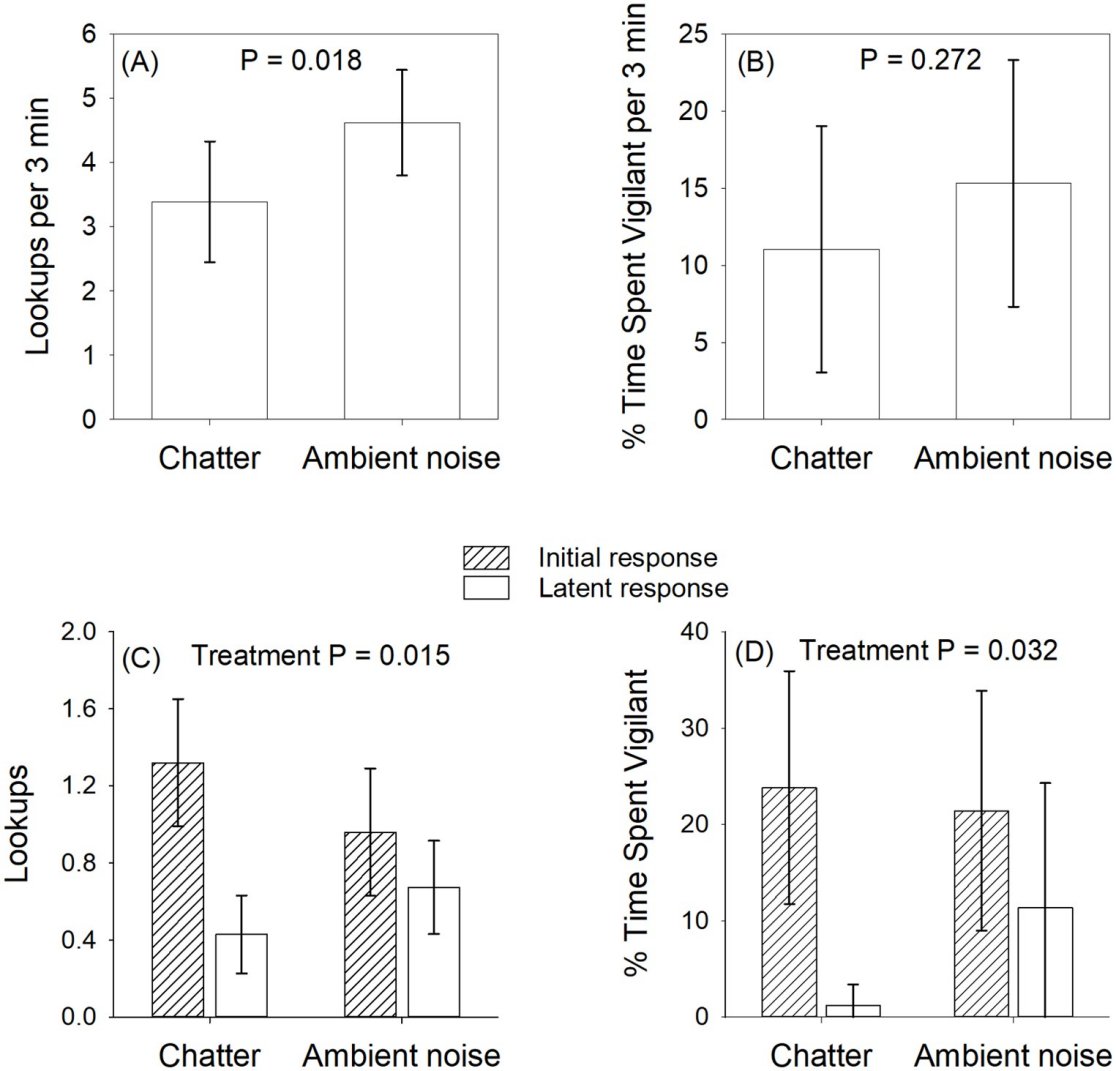
Effect of bird chatter on squirrel vigilance behaviors. (A) Estimated mean number of lookups by squirrels (controlling for habitat vulnerability and initial response to hawk call) when exposed to either chatter or ambient noise during the 3 min treatment period following the presentation of a hawk call. (B) Percent of time spent vigilant during the 3 min treatment period, controlling for habitat vulnerability and initial response to hawk call. (C) Change in number of lookups per 30 sec between the post-hawk period and the final minute of the 3 min treatment period (means not adjusted for effects of habitat vulnerability). (D) Change in percent time spent vigilant between the post-hawk period and the final minute of the 3 min treatment period (means not adjusted for effects of vulnerability). Error bars represent 95% confidence intervals.

Squirrels exposed to chatter exhibited significantly fewer lookups during the 3-minute treatment period than those exposed to ambient noise (P = 0.018; Table 2; Fig 2A). Likewise, the decline in number of lookups following the hawk call was significantly greater in squirrels exposed to chatter than those exposed to ambient noise (P = 0.015; Table 2; Fig 2C). We detected no difference in the overall amount of time spent vigilant during the treatment period (P = 0.272; Table 2; Fig 2B), but squirrels exposed to chatter exhibited a significantly greater decline in time spent vigilant following the hawk call than squirrels exposed to ambient noise (P = 0.032; Table 2; Fig 2D).

**Table 2:** Results of permutation tests of linear models predicting squirrel vigilance as a function of treatment (ambient noise vs chatter), vulnerability (in tree vs on ground), and vigilance level immediately following a hawk call but prior to the onset of the treatment playback.

Response	Effect	Estimated slope^c^	Iterations	P
Number of lookups^a^	Treatment	-1.11	5000	0.018
	Vulnerability	2.59	2812	0.035
	# post-hawk lookups	2.07	5000	0.0004
Change in number of lookups^b^	Treatment	-0.17	5000	0.015
	Vulnerability	0.08	69	0.594
	# post-hawk lookups	-0.75	5000	< 0.0001
Percent of time vigilant^a^	Treatment	-2.76	268	0.272
	Vulnerability	-3.32	161	0.385
	% post-hawk vigilance	0.66	5000	< 0.0001
Change in percent of time vigilant^b^	Treatment	-5.67	2996	0.032
	Vulnerability	2.99	51	0.94
	% post-hawk vigilance	-0.63	5000	< 0.0001

^a^ Number of lookups and Percent of time vigilant were measured over the entire 3 min treatment period.

^b^ Change in these responses is a comparison of responses during the 30 s post-hawk period and the final minute of the treatment period; both measures are standardized per 30 s period.

^c^ Negative slopes for treatment indicate lower vigilance by squirrels in the chatter treatment; negative slopes for vulnerability indicate lower vigilance by squirrels on the ground.

### Variation among playback exemplars

**Fig 3:**
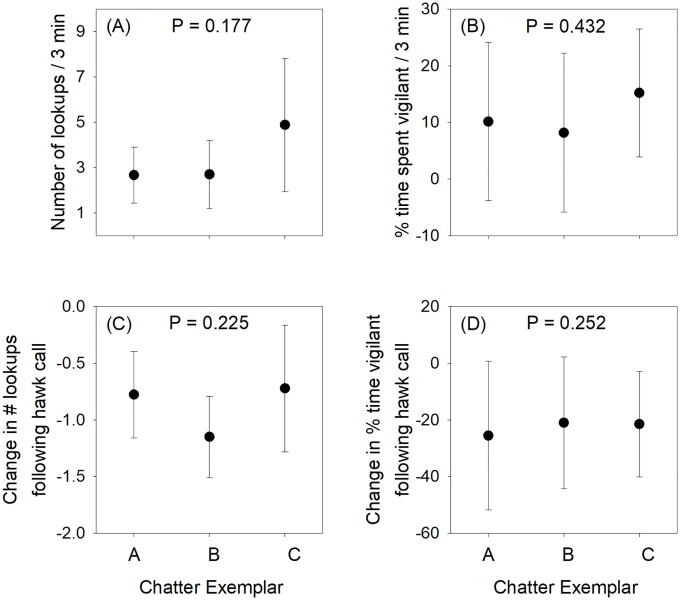
Vigilance responses of gray squirrels to three different exemplar recordings of bird chatter (see Table 1 for list of species on each track). Means and 95% confidence intervals are estimated from permuted ANOVAs controlling for habitat vulnerability and initial response to the hawk call. (A) Number of lookups during the 3 min treatment period. (B) Percent of time spent vigilant during the 3 min treatment period. (C) Change in number of lookups per 30 sec between the post-hawk period and the final minute of the 3 min treatment period. (D) Change in percent time spent vigilant between the post-hawk period and the final minute of the 3 min treatment period. Error bars represent 95% confidence intervals. No differences in any panel are statistically significant.

Squirrels did not differ in their response to the three chatter exemplars when controlling for vulnerability and vigilance behavior during the post-hawk period (permutation tests of ANOVA models; number of lookups, P = 0.177, Fig 3A; percent time spent vigilant P = 0.432, Fig 3B; N = 9 trials for playback exemplar Chatter_A, 10 trials for Chatter_B, and 9 trials for Chatter_C), nor did the change in lookups or vigilance over time differ among the exemplars (change in lookups, P = 0.225, Fig 3C; change in percent time spent vigilant, P = 0.252; Fig 3D). Similarly, squirrels did not differ in those same measures in response to the three ambient noise exemplars when controlling for vulnerability and vigilance behavior during the post-hawk period (all P > 0.173; N = 10 trials for exemplar Ambient_A, 8 trials for Ambient_B, and 8 trials for Ambient_C).

## Discussion

Here we show that eavesdroppers may extract information about the safety of the environment from heterospecific non-alarm auditory cues from species with which they do not intimately associate. Gray squirrels exposed to bird chatter expressed significantly lower and more rapidly declining levels of vigilance behavior than those exposed to ambient noise, suggesting they used information contained in bird chatter as a cue of safety. Bird chatter, comprising contact calls and other non-alarm sounds emanating from multiple individuals of multiple species, is likely to indicate safety because such sounds are generally given when imminent threat has not been detected [22]. Although some species attend particularly to species that serve as “community informants” within mixed-species flocks, gray squirrels do not travel or otherwise directly associate with mixed species bird flocks, supporting the hypothesis that they eavesdrop on non-alarm auditory cues of species with which they do not closely associate for indicators of safety.

Although many studies have investigated heterospecific eavesdropping on alarm calls as indicators of threat, few studies have investigated non-alarm signals that eavesdroppers might use as indicators of safety [4,41–43]. ‘Sentinal’ calls are the most well studied indicators of safety. Individuals of some species act as sentinels for a foraging group, keeping a lookout and in the absence of danger providing ‘sentinel calls’ which elicit lower vigilance behavior from listeners; ‘all-clear’ calls similarly indicate that a predator threat has passed or that an alarm call was false [7,10,11,13,18,19,44]. A few studies have demonstrated eavesdropping on non-sentinel calls within co-foraging groups as an index of safety [9,17,20,45]. However, to our knowledge, our study is the first to demonstrate eavesdropping on non-sentinal calls outside of a co-foraging group association [9,41,43], indicating that eavesdropping on cues of safety may be more widespread than previously recognized.

Recognition of bird chatter as a sign of safety is likely adaptive, as squirrels that can safely reduce their vigilance level in the presence of bird chatter presumably are able to increase foraging success [13]. A forager’s optimal level of vigilance can be influenced by the level of vigilance presented by other prey that share the same predators in a given community [4,6]. Some members of a community, such as birds, are better at detecting predators than others due to sensory and ecological differences [43,46,47]. In turn, other members of the community that share the same predators, such as small mammals, may rely on those species for indicators of threat [4]. Although seemingly adaptive, the changes in vigilance in response to chatter that we observed raise interesting questions regarding how squirrels acquire the ability to recognize contact calls from a variety of species with which they don’t intimately associate. One possibility is that individual squirrels may learn to recognize correlations between particular vocalizations of other species in the community and the presence or absence of their shared predators, similar to how individuals of some species learn alarm calls [4,48–50]. Alternatively, attention to generalized auditory cues of safety could be favored by selection if individuals that are able to recognize when an environment is safe in turn maximize their energetic expenditure towards foraging and therefore have higher reproductive success, leading to this trait becoming innate over evolutionary time [1,27,51]. This latter hypothesis assumes that auditory components of chatter are consistent enough across species that they are transferable across ecological communities.

In our study, squirrels significantly lowered their vigilance level in the presence of bird chatter. However, it is possible that the squirrels lowered their vigilance level in response to specific species present in the chatter recordings rather than in response to the chatter in its entirety [10,19]. For example, Hetrick and Sieving’s work [15] suggests that tufted titmice function as community informants because in addition to producing alarm calls in the presence of predators, titmice also use abundant contact calls throughout the day that may encode information about perception of threat. Additionally, some bird species exhibit lower vigilance levels when foraging in mixed flocks that include titmice than when foraging alone or in mixed flocks that do not include titmice [9,15,45]. Thus, if all of our chatter recordings contained titmice contact calls, squirrels may have cued in on a particularly informative species that is common enough in their habitat to constitute an “intimate,” if highly indirect, ecological association. However, we detected titmice calls on only one of the three chatter exemplars we used in playback experiments, yet we detected no significant differences in squirrel response among the three exemplars. Only one bird species, dark-eyed junco, was detected in all three exemplars, so it is possible that squirrels were cuing in on contact calls of a single species. However, juncos are patchily distributed and temporally ephemeral on our study site. Hence, it seems unlikely to us that squirrels would rely exclusively upon juncos to obtain useful information about safety. Our understanding of bird chatter as a heterospecific safety cue would benefit from future studies of vigilance responses of gray squirrels or other eavesdroppers to playback treatments that better control the species contributing to chatter. Interestingly, all three of our chatter exemplars contained wing flutter noises and noises arising from foraging birds hopping in dry leaves. Though perhaps unlikely, we cannot exclude the possibility that squirrels in our study were cuing in on these other sounds, which potentially could indicate a group of birds that is foraging as if they perceive a relatively low predation threat (e.g., [52]). Studies that clearly isolate the contributions of vocal and non-vocal cues could improve our understanding of the reliability of chatter as an index of safety.

In addition to vocal and non-vocal sounds produced by birds, our recordings contained other noises that potentially could influence squirrel behavior. For example, all three chatter exemplars contained wind noise and noise generated by the building near the recording site, and some exemplars contained distant traffic and other noises. Similarly, all three contained a greater or lesser level of white noise and clipping sounds, which could have startled squirrels when the recordings were played in the field. We would expect such sounds to increase squirrel vigilance by either startling them or by masking natural biotic cues of safety to which they attend (thereby forcing them to be more vigilant than they would be if they had access to those biotic cues). Such noises therefore should *reduce* our ability to detect an overall effect of chatter on squirrel vigilance behavior and thereby drive the observed patterns in a direction opposite to that predicted by the hypothesis that chatter functions as a cue of safety. However, we still detected strong and statistically significant evidence that exposure to chatter reduces squirrel vigilance. Moreover, most of these abiotic sounds (other than clipping noise) also were present in the ambient noise treatment exemplars, thereby controlling for the effects of abiotic background noise on the recordings. In sum, we find no evidence that suggests our conclusions could be attributed to abiotic noises on our playback recordings.

In addition to recognizing indirect cues about the safety level of the environment, we found that squirrels responded to direct cues of danger—hawk call playbacks—by significantly increasing the time they spent engaged in vigilance behavior as well as number of times they looked up during otherwise non-vigilance behaviors. This response indicates that they were primed to be vigilant to the possibility of predators in the area prior to the treatments of chatter or ambient noise. Although some studies have indicated that some terrestrial species respond more strongly to overheard information about predator risk than to the actual predator [3,53,54], the increase in vigilance after the hawk call playback observed in our study supports the hypothesis that prey respond to direct information about risk of predation [4,32,55].

Over the past decade, anthropogenic noise levels have steadily increased with varying effects on different ecosystems, presenting a concern for conservation efforts [33,56–59]. While our study does not directly address the issue of noise pollution, it identifies a novel component of information networks that anthropogenic noise might cover up [44]. Indeed, relatively quiet chatter noises may be more susceptible to interference from anthropogenic noise than loud alarm calls. If bird chatter were masked by anthropogenic noise, this publicly available safety cue could be lost to the network of eavesdroppers. The lack of safety signals might cause squirrels and other eavesdroppers to allocate more energy towards vigilance behaviors and less towards foraging, potentially compromising fitness [4,33,57,58].

Overall, our study provides further insight into the complexity of information utilized by eavesdropping species and adds to our understanding of the role of noise and eavesdropping in behavioral ecology.

## Supporting information

S1 Data Data used in Lilly et al. "Eavesdropping Squirrels Infer Safety from Bird Chatter". Worksheet 1 provides definitions of variables used in each data set. Worksheet 2 provides data pertaining to squirrel responses to the red-tailed hawk call playbacks used to prime squirrels in the experiment. Worksheet 3 provides data pertaining to squirrel responses to the treatment playbacks, and comparisons of pre- and post-treatment responses.
(XLSX)
